# Analysis of Jumping-Landing Manoeuvers after Different Speed Performances in Soccer Players

**DOI:** 10.1371/journal.pone.0143323

**Published:** 2015-11-24

**Authors:** Abdolhamid Daneshjoo, Noor Azuan Abu Osman, Mansour Sahebozamani, Ashril Yusof

**Affiliations:** 1 Department of Sports Injuries and Corrective Exercises, Faculty of Physical Education and Sport Science, Shahid Bahonar University of Kerman, Kerman, Iran; 2 Department of Biomedical Engineering, University of Malaya, Kuala Lumpur, Malaysia; 3 Sports Centre, University of Malaya, Kuala Lumpur, Malaysia; Bern University of Applied Sciences, SWITZERLAND

## Abstract

**Purpose:**

Running at high speed and sudden change in direction or activity stresses the knee. Surprisingly, not many studies have investigated the effects of sprinting on knee’s kinetics and kinematics of soccer players. Hence, this study is aimed to investigate indices of injury risk factors of jumping-landing maneuvers performed immediately after sprinting in male soccer players.

**Methods:**

Twenty-three collegiate male soccer players (22.1±1.7 years) were tested in four conditions; vertical jump (VJ), vertical jump immediately after slow running (VJSR), vertical jump immediately after sprinting (VJFR) and double horizontal jump immediately after sprinting (HJFR). The kinematics and kinetics data were measured using Vicon motion analyzer (100Hz) and two Kistler force platforms (1000Hz), respectively.

**Results:**

For knee flexion joint angle, (p = 0.014, η = 0.15) and knee valgus moment (p = 0.001, η = 0.71) differences between condition in the landing phase were found. For knee valgus joint angle, a main effect between legs in the jumping phase was found (p = 0.006, η = 0.31), which suggests bilateral deficit existed between the right and left lower limbs.

**Conclusion:**

In brief, the important findings were greater knee valgus moment and less knee flexion joint angle proceeding sprint (HJFR & VJFR) rather than no sprint condition (VJ) present an increased risk for knee injuries. These results seem to suggest that running and sudden subsequent jumping-landing activity experienced during playing soccer may negatively change the knee valgus moment. Thus, sprinting preceding a jump task may increase knee risk factors such as moment and knee flexion joint angle.

## Introduction

Soccer is a sport that needs intermittent, non-continuous exercises which includes many sprints of different intensities especially during a match [1, 2]. Commonly, sprint performance is measured as speed; which is described as quotient of distance divided by time, where distance represents the actual length covered [3]. Previous research has shown that speed is a key component in playing soccer especially at a high level [2]. Unfortunately, speed has also been associated with injury and classified as an intrinsic risk factor [4, 5]. Wong and Hong (2005) in their review on soccer related lower extremity injury, reported 21 studies which showed higher injury rates during competition than training [6]. In other words higher speed of play during competitions may exert more loads or forces on the knee joint, which in turn may raise frequency of injury.

The knee is one of the most commonly injured sites in soccer [7, 8]. On average in the United States, each year, there are about 75000 anterior cruciate ligament reconstructions which costed around $25000, and over a 10-year period the amount has exceeded $18.7 billion [9]. Some studies distinguished mechanisms for knee injuries as contact and non-contact [4, 5]. Although, due to robustness of the game one may think that contact injury occurs often, the fact is, most knee injuries in soccer players is the non-contact injury involving planting, pivoting, or landing [10–12]. A smaller amount of knee-flexion joint angle (within 10–30°) [13–15], greater knee-valgus joint angle, and greater vertical and posterior ground reaction forces during landing increase knee joint loading and injury risks [16, 17]. Furthermore, a study reaffirms that quadriceps exert its maximum anterior shear force when knee flexion joint angles are within 10 to 30° [15]. An erect landing posture and less knee flexion joint angle result in higher ground reaction forces (GRF) than a more flexed landing posture, could consequently lead to knee injury [16, 17]. High knee valgus joint angle and load during jumping-landing maneuvers are reported as important predictors of non-contact anterior cruciate ligament injury risk [18, 19].

Other determining factor of knee injury is kinetic and kinematic asymmetry between two legs [20]. Van der Harst and colleagues (2007) found increased knee flexion after landing on dominant leg compared to non-dominant leg among athletes (soccer, baseball, korfball and Judo). However, they reported no significant differences in the kinetic variables (ground reaction forces and moment) between legs. In sports with asymmetric kinetic patterns like soccer, players frequently use one leg for ball kicking, cutting and landing skill [21] and this can cause of differences in the muscle strength and flexibility [21], and pelvic and knee angles [22] between the two legs. Generally, identification of bilateral asymmetries is useful in clinical exercises for comparative and corrective purposes [21, 23, 24].

It is unknown whether sprint activity followed by plyometric and explosive activity ie jumping and landing, would change the kinetic and kinematics variables of soccer players. Understanding the etiology and mechanism of injury is a critical step in injury prevention research [5]. To address the issue on prevention of knee injuries among soccer players and design proper prevention program, alterations in kinetic and kinematic parameters could be used as a reference [25]. Therefore, the main purpose of the present study was to analyze jumping-landing maneuvers after different run-up speeds using related kinetics (GRF and knee valgus moment) and kinematics (knee flexion and valgus joint angles) on risk factor measures of male soccer players. The results of this study would provide information on the impact of sprint running on kinetics and kinematics and bilaterally differences of the knee in soccer players. It was hypothesized that higher running speed would augment jumping-landing forces hence increase vulnerability to knee injury.

## Methods

### Ethics Statement

This study was approved by the ethical committee of Sports Centre Research Committee at University of Malaya. All the participants were informed orally about the procedures they would undergo and their written consents were taken (S1 Appendix).

### Participants

Twenty-three male soccer players (20–24 years) were recruited from University of Malaya soccer team. The players inclusion criteria were: had almost daily training at least five years’ experience of playing soccer, and without history of major lower limb injury or disease. Descriptive data (mean ± SD) for the participants were; age 22.1 ± 1.7 years, body mass 64.1 ± 7.1kg; height 168.7 ± 7.6 cm; leg length (right = 885 ± 38.5 left = 885 ± 38.7 mm), knee width (right = 97.8 ± 6.8 left = 97.5 ± 6.3 mm) and ankle width (right = 66.6±4.6 left = 67.0 ± 4.6 mm).

### Kinetic and kinematic tests

Movement was recorded using Vicon motion analysis system (100Hz; MX Oxford Metric; Oxford, UK) with five cameras (T40s), and two Kistler force platforms (1000Hz; type 28112A2-3S, Kistler holding AG, Switzerland) which were embedded in the floor and in the middle of the capture volume. Motion analysis system and force platforms were synchronized and calibrated prior to testing. VICON plug-in gait was used to process motion capture and ground reaction force data. In order to capture the movement, 16 reflective markers (according to Helen-Hayes) were affixed to bony landmarks of the lower limb. Markers were attached with adhesive tape or Velero straps directly to the participant's skin or footwear. All the markers were attached by the same researcher each time. Anthropometric data of all the participants were obtained prior to testing. Kinematic and kinetic data were filtered using a fourth- order zero-lag Butterworth 12-Hz low-pass filter automatically.

Prior to data collection, each subject performed a 5-min dynamic warm-up including series of submaximal vertical jumps. The examiner determined the dominant leg of the subjects by asking them which leg they would prefer for kicking the ball. Before testing, each subject was shown the maneuvers and allowed to perform each task to familiarize with the actual tests. They performed 4 tasks; vertical jump (VJ), vertical jump after slow running (VJSR), vertical jump after fast running (VJFR) and double horizontal jump after fast running (HJFR).

**Fig 1:**
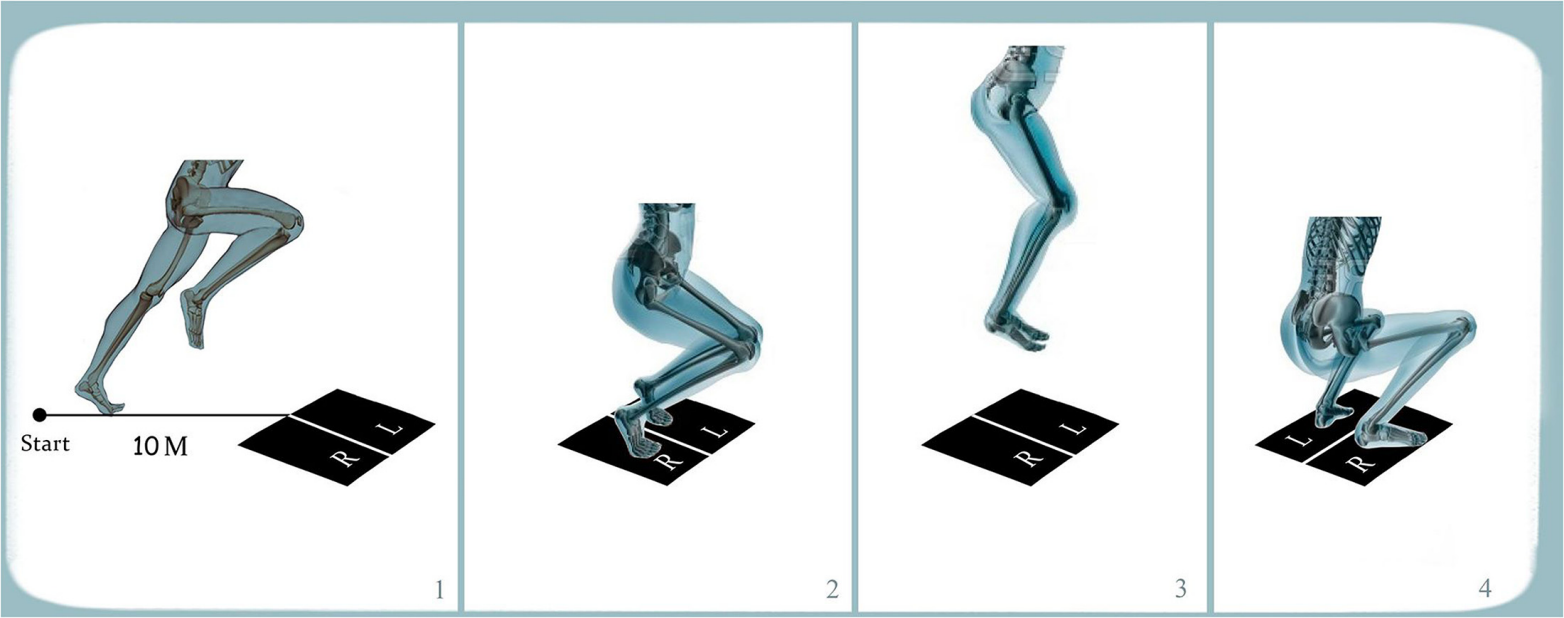
Vertical jumping and landing after 10 m slow and fast run. The subjects run 10m at speed below (VJSR) and above their average speed (VJFR) and perform a vertical jumping and landing while was on the force plate.

**Fig 2:**
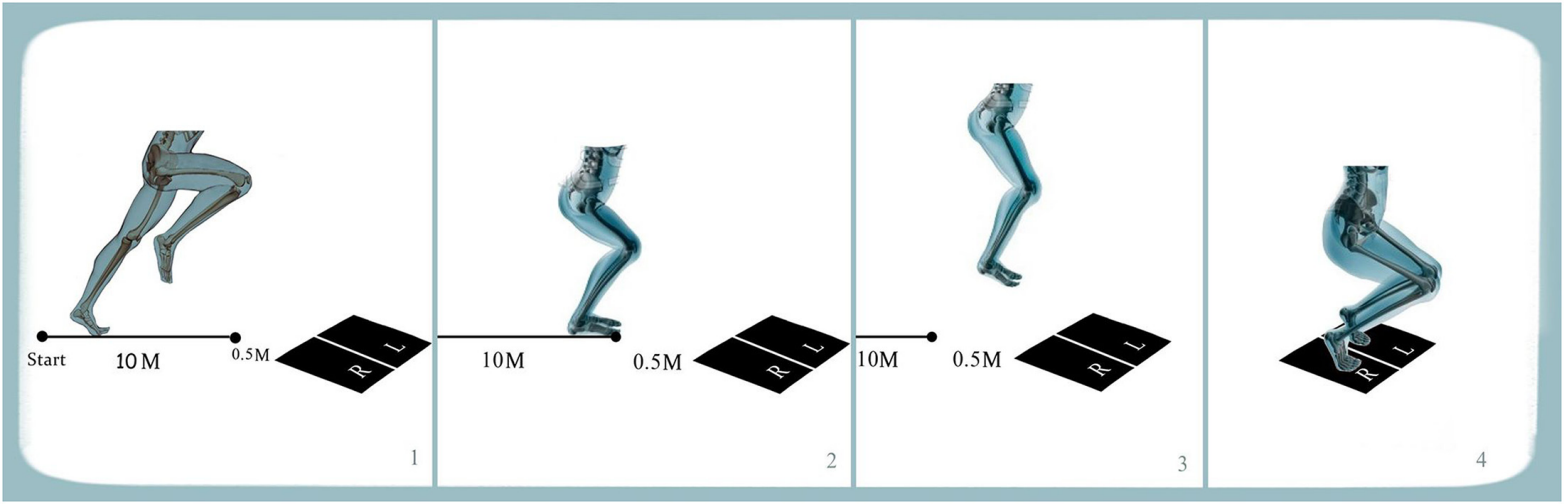
Double horizontal jump after 10m run (HJFR). The subjects run 10m and carried out double horizontal jump before reaching the force plate and landed on the force plates.

The subjects were instructed to perform a two-foot vertical jump-and-land when contacting the force plates with both feet (conditions VJ, VJSR, VJFR). The 2 force platforms were positioned that each foot would contact one different platform fully and separately as presented in Fig 1. With regard to the vertical jump while running slowly, the subjects were instructed to run for 10m at a speed lower than 60% of their maximum speed and to perform a vertical jump, taking off from the force plate and landing back on it. While in the next condition (condition VJFR) the subjects were asked to run at their maximum speed in 10m and perform jumping and landing on the force plate (Fig 1). In condition HJFR, subjects carried out double horizontal jumps after fast running off the force plate and landed on the force plates (Fig 2). We have chosen these conditions because they mimic real soccer match situation. Three trials were recorded for all participants in each condition and the best performances (The criteria used were speed test and the data collected from all cameras) from two phases of jumping and landing while the feet were in contact on force plate was used for analyses. The trial with the most complete camera data for the VJ condition was chosen for analysis.

Joint moments are calculated using an inverse dynamics approach, where GRF (force plate data) and segment accelerations (marker data; the reflective markers attached on the participant) are combined [26, 27]. GRF data has been identified in present study as an important joint loading parameter associated with development of injuries [28]. GRF was normalized to body mass (%BM), and knee valgus moment was normalized to the body mass and height (%BM*H). Peaks of GRF and knee valgus moment were calculated for all the participants. Kinematic and kinetic variables of interest were knee angles in sagittal and frontal plane and peak knee valgus moments in the frontal plane in the jumping and landing phases [27].

To compare the speed of the subjects with their speed during the kinetic and kinematic tests, the subjects were asked to perform 3 times the 10m test, with a 3-min recovery period in between. The 10-meter speed test has been shown to be a reliable test in measuring the soccer players’ running speed (ICC = 0.85) [29]. The players started from a standing position, and the timing system was triggered as soon as they left the starting mat. The time spent in this test was recorded (with a resolution of 0.01 s) using a handheld stopwatch (Sportline 240, Sportline,Inc, Hazleton, Pa) which was operated by one of the researchers. The minimum score (i.e. fastest time) was defined as the best score and used for the analysis and comparison with the records of VJFR and HJFR conditions. This test was performed prior to the kinetic and kinematic test to determine what actual speed record was. In this research slow speed is defined as running speed of less than 60% of the maximum speed.

### Statistical analysis

To compare the variables between the four conditions (VJ, VJSR, VJFR, HJFR) and legs (dominant and non-dominant), the 4 ×2 (condition vs leg) repeated measures ANOVA was used. The Bonferroni’s post hoc test for multiple comparisons was used. The Kolmogorov-Smirnov (KS) statistic was employed for assessing normality of the distribution of scores (p>0.05). The effect sizes of each variable were tested using partial eta (η) squared (0.01 = small effect, 0.06 = medium effect, and 0.14 = large effect), [30, 31]. The alpha level was set at 0.05 for all comparisons.

## Results

Descriptive results of the speed test and running speed during kinetic and kinematics were 10m sprint (s):1.76 ± 0.03; slow sprint during testing (condition VJSR) (s): 2.34 ± 0.06; fast sprint during testing (condition VJFR) (s):1.82 ± 0.1. The analysis did not show significant difference between fast sprint before testing and fast sprint during condition VJFR (t = 1.76, p = 0.09) and condition HJFR (t = 1.13, p = 0.27).

The mean and standard deviation of knee joint angles, peak GRF and knee valgus moments are presented in Table 1. Here the jumping and landing phases are described separately.

**Table 1:** Average (±SD) knee joint angles, GRF (%BW) and knee valgus moments (%BW*H) in jumping and landing phase (degrees).

	Peak knee valgus angle (°)		Peak knee flexion angle (°)		Peak GRF (%body mass)		Peak moment (%BM*H)	
	Non-dominant leg	Dominant leg	Non-dominant leg	Dominant leg	Non-dominant leg	Dominant leg	Non-dominant leg	dominant leg
**Jumping phase**								
VJ	94.90 ± 8.4 ^a^	85.14± 6.4	23.49± 4.1	20.34± 2.1	47.48± 32.7	13.58± .7	3.71± .4	4.07±.5
VJSR	83.96± 3.9 ^a^	88.35± 3.5	16.65± 4.3	17.10± 2.9	14.50± 0.6	14.19± .8	4.10± .5	6.31±.9
VJFR	76.35± 12.2	70.08± 11.3	4.89± 6.1	12.96± 4.3	38.13± 23.1	14.81± 1.0	6.02± .9	6.11±.9
HJFR	96.76± 5.9	95.05± 7.9	21.46± 6.3	14.18± 6.9				
**Landing phase**								
VJ	103.52± 12.3	74.18± 7.1	35.35± 9.0 ^d^	21.32± 3.2	30.83± 2.6	25.72± 1.8	7.54± 1.0 ^c^ ^,^ ^d^ ^,^ ^e^	7.71± 1.1
VJSR	104.10± 7.1	88.30± 6.5	23.40± 6.5	20.20± 2.7	43.29± 13.0	29.82± 2.5	9.99± 1.9 ^b^ ^,^ ^e^	9.95± 1.4
VJFR	88.86± 13.0	86.13± 13.3	15.6± 5.7 ^b^	14.23± 4.2	65.76± 36.4	35.08± 4.2	9.66± 1.6 ^b^ ^,^ ^e^	11.85± 1.9
HJFR	103.97± 12.5	94.79± 7.2	23.02± 6.0	16.31± 5.6	87.24± 45.4	34.96± 2.5	13.47± 2.2^b^ ^,^ ^c^ ^,^ ^d^	13.91±2.5

° = degree; SD = standard deviation; BM = body mass; H = height; VJ = double vertical jump; VJSR = double vertical jump after slow running, VJFR = double vertical jump after fast running; HJFR = double horizontal jump after fast running.

^a^ = significant differences between legs;

^b^ = significant differences with VJ;

^c^ = significant differences with VJSR;

^d^ = significant differences with VJFR;

^e^ = significant differences with HJFR.

### Kinetics and kinematics of jumping phase

For knee flexion joint angle no differences were found between conditions (F_3,19_ = 0.98, p = 0.425) and leg dominance. For knee valgus joint angle, results showed significant main effect between leg dominance (F_1,21_ = 9.34, p = 0.006, η = 0.31) but no difference was found between the four conditions. The differences of knee valgus joint angle between legs were found in conditions VJ (p = 0.024) and VJSR (p = 0.040). This result suggested existence of different knee valgus joint angle between right and left lower limb in this phase. No significant differences were found in knee valgus moment and GRF between conditions (p>0.05).

### Kinetics and kinematics of landing phase

For knee flexion joint angle, the results showed significant differences between the four conditions (F_3,19_ = 3.80, p = 0.014, η = 0.15) with large effect size (0.15) in the landing phase. The Bonferroni post-hoc showed significant differences between conditions VJ with VJFR (p = 0.048). Mean of Knee flexion joint angle in VJFR was found lesser (dominant leg & non-dominant leg; 15.6±5.7°, 14.23± 4.2°) than VJ condition (dominant leg & non-dominant leg; 35.35±9.0°, 21.32± 3.2°).

For knee valgus moment in the landing phase, the results showed significant differences between conditions (F_3,19_ = 15.19, p = 0.001, η = 0.71) with large effect size (0.71). The Bonferroni post-hoc test showed significant pair-wise difference between these conditions; VJ and VJSR (p = 0.004), VJ and VJFR (p = 0.005), VJ and HJFR (p = 0.001), VJSR and HJFR (p = 0.001), and VJFR with HJFR (p = 0.001). The greater valgus moment was found in HJFR (dominant leg & non-dominant leg 13.47± 2.2, 13.91±2.5%BM*H) than other conditions, while the smaller knee valgus moment indicated in VJ (dominant leg & non-dominant leg 7.54± 1.0, 7.71± 1.1%BM*H) than other conditions. Relatively greater knee valgus moment seen after landing following fast running (HJFR) has higher predisposition to knee injury due to greater force transfer to the knee.

For knee valgus joint angle, analysis did not show any difference between the four conditions, and leg dominance in the landing phase (p>0.05). In addition, in all indices, no differences were found in leg dominance and GRF.

## Discussion

The main aim of this study was to investigate the effects of sprinting at different speed and subsequent jumping-landing maneuvers on knee injury risk factors such as knee angle in frontal and sagittal planes, GRF and knee valgus moment of male soccer players. The main finding in this study was a greater knee valgus moment during landing phase in fast sprint (HJFR) rather than other conditions. A number of reports have shown that most sports-related ACL injuries occur during non-contact situations [15, 32]. Landing with greater knee valgus moment appears to increase knee joint load which can amplify risk of injury [33]. It has been shown that the increased proximal tibia anterior shear force and knee valgus moment during landing enhance the non-contact ACL injury risk [34, 35]. According to Hewett et al. (2006) ACL-injury status is predictable by knee valgus moment with 73% specificity and 78% sensitivity [33]. In this study, the greater valgus moment was found in HJFR, while lesser knee valgus moment was observed in VJ. As the speed and intensity increase, soccer players tend to have less body control [6] which may increase the chances of injury. Minimizing knee valgus moments during soccer appears to be crucial in the prevention of ACL injuries [18]. Hence, it is suggested that players should be encouraged to minimize HJFR during playing soccer to reduce knee valgus moment and consequently minimize their risk of ACL injury.

The findings of this study showed less knee flexion joint angle (peak) during fast sprint (VJFR) compared to vertical jump in the landing phase. ACL loading, resulting from a constant anterior shear force increased as the knee flexion joint angles decreased which may in turn present an increased risk factor for ACL injuries [36]. Wang (2011) describes the knee flexion joint angle as an important kinematic factor during the landing phase as it is highly relevant to how force is affected [37]. Moreover, the greater knee valgus moment and less flexion joint angle during fast sprint may be associated with the higher incidence of knee injury and especially ACL injury in male soccer players.

The absence of a significant difference in knee flexion joint angle between the conditions VJFR & HJFR in both jumping and landing phase is a novel finding suggesting that trained athletes may perform VJFR and HJFR with similar knee flexion joint angle. Nagano et al. (2007) using computer simulation found that the magnitude of knee flexion joint angle was similar between horizontal and vertical jumps [38]. In contrast, Fukashiro and colleagues (2005) reported that joint kinematics of the hip, knee and ankle were similar between standing horizontal jump and standing vertical jump during push-off. They reported that knee flexion angle in the standing horizontal jump was 10° less than standing vertical jump among eight adult male Australian Football players, but the difference was not statistically significant [39]. The horizontal and vertical jumps have some differences in the jumping phase and similarities in the landing phase. The horizontal jump involves both vertical and horizontal propulsive forces in jumping phase [40]. Moreover, body's center of mass needs to be located above the feet during a vertical jump, whereas this requirement is not so strict during a horizontal jumping phase [38]. In this study, following a 10m run, subjects performed both the horizontal and vertical jumps which involved muscle’s stretch-shortening cycle. This difference could be a contributing factor in the lack of changes observed in the knee flexion angle. This could be attributed to years of training; trained players are naturally exposed to double horizontal jumps (condition HJFR) activities more than vertical jump (condition VJFR), resulting in greater familiarization and soft jumping-landing.

Greater knee adduction in non-dominant leg rather than dominant leg was found which suggests existence of bilateral different knee valgus joint angle. Usually, the non-dominant leg provides postural support while the dominant leg is used to kick the ball [21, 41]. In soccer with asymmetric kinetic patterns, more emphasis is given to one side of the legs which negatively influence muscle balance [42]. Soccer players almost never use both legs with equal emphasis [43]. Soccer players’ preference to use one side more than the other [21].is related to hemispheric dominance of the brain in the opposite side. This is the possible cause for this result in professional soccer players

## Conclusion

In conclusion the main findings were greater knee valgus moment and less knee flexion joint angle in fast sprint (HJFR, VJFR) rather than no sprint condition (VJ) in landing phase. The knee flexion joint angle in VJFR was found to be significantly less than in the VJ condition. Significant differences between VJ with VJFR and HJFR in the knee valgus moment were found, while the greater valgus moment was found in HJFR and lesser knee valgus moment in VJ. The present results support the theory that fast sprint increases knee valgus moment during landing in soccer players. We infer that higher knee valgus moment and less knee flexion joint angle could result from the running fast among soccer players during the landing phase which may present as increased risk factors for knee injuries. The findings of this research may be helpful for coaches and trainers who can strategize training programs to reduce knee injury risk factors of male soccer players as well as reducing bilateral different knee valgus joint angle. These results affirm the physical performance and movement pattern experienced during soccer may negatively change the knee valgus moment.

## Supporting Information

S1 Appendix Statement of Consent. (DOC)
