# Correction: Analysis of Jumping-Landing Manoeuvers after Different Speed Performances in Soccer Players

**DOI:** 10.1371/journal.pone.0152630

**Published:** 2016-03-24

**Authors:** Abdolhamid Daneshjoo, Noor Azuan Abu Osman, Mansour Sahebozamani, Ashril Yusof

There is an error in the Acknowledgements of the published article. The Acknowledgements should read:

This study was supported by UM/MOHE/ HIR Project No. D000014-16001. The authors are grateful to Dr. Mohammadtaghi Amiri-Khorasani for expert guidance during the early stage of this project and Dr. Jos Vanrenterghem for his valuable comments. The authors also acknowledge the expert guidance of Nafiseh Khalaj, who contributed to biomechanics data collection and manuscript preparation (related to data collection).
