# Supplementary material for: Analysis of Jumping-Landing Manoeuvers after Different Speed Performances in Soccer Players
Source: PLoS One. 2015 Nov 24;10(11):e0143323. doi: 10.1371/journal.pone.0143323 (PMC4658049; doi:10.1371/journal.pone.0143323)
Supplement: S1 Appendix — (DOC) [file pone.0143323.s001.doc]

**Statement of Consent**

I am willing and give my consent to participate in the research titled “analysis of jumping-landing manoeuver after different speed performances in soccer players”. I have seen and read the material to be published. I have discussed this consent form with ABDOLHAMID DANESHJOO, who is an author of this paper. I agree to my profile and comments being included in the data collection on the understanding that confidentiality and privacy will be maintained. I am also aware that my name will not appear on any documentation and my identity will not be disclosed at any point in the research or data analysis and, thus, I will remain completely anonymous.

I understand that under the license which the PLoS uses (the Creative Commons Attribution License) material published in PLoS journals can be redistributed freely and used for any legal purpose, including translation into other languages and commercial uses. I also understand that signing this consent form does not remove my rights to privacy.

Name _________________________________

Date _________________

Signed _________________________________

Author _________________________________

Date _________________

Signed _________________________________
